# Variation in Community Structure of the Root-Associated Fungi of *Cinnamomum camphora* Forest

**DOI:** 10.3390/jof8111210

**Published:** 2022-11-15

**Authors:** Deqiang Chen, Jiaoyan Zeng, Xiaohui Wan, Yonglong Wang, Siren Lan, Shuangquan Zou, Xin Qian

**Affiliations:** 1 College of Forestry, Fujian Agriculture and Forestry University, Fuzhou 350002, China; 2 Fujian Colleges and Universities Engineering Research Institute of Conservation and Utilization of Natural Bioresources, College of Forestry, Fujian Agriculture and Forestry University, Fuzhou 350002, China; 3 Key Laboratory of National Forestry and Grassland Administration for Orchid Conservation and Utilization, College of Landscape Architecture, Fujian Agriculture and Forestry University, Fuzhou 350002, China; 4 Large Data Institute, Fuzhou University of International Studies and Trade, Fuzhou 350002, China; 5 Fujian Forestry Investigation and Planning Institute, Fuzhou 350002, China; 6 Faculty of Biological Science and Technology, Baotou Teacher’s College, Baotou 014030, China; 7 College of Life Sciences, Fujian Agriculture and Forestry University, Fuzhou 350002, China

**Keywords:** fungal communities, *Cinnamomum camphora*, root-associated microorganisms, slope positions

## Abstract

Plant-associated microbial communities play essential roles in the vegetative cycle, growth, and development of plants. *Cinnamomum camphora* is an evergreen tree species of the Lauraceae family with high ornamental, medicinal, and economic values. The present study analyzed the composition, diversity, and functions of the fungal communities in the bulk soil, rhizosphere, and root endosphere of *C*. *camphora* at different slope positions by high-throughput sequencing. The results showed that the alpha diversity of the fungal communities in the bulk soil and rhizosphere of the downhill plots was relatively higher than those uphill. A further analysis revealed that Mucoromycota, the dominant fungus at the phylum level, was positively correlated with soil bulk density, total soil porosity, mass water content, alkaline-hydrolyzable nitrogen, maximum field capacity, and least field capacity. Meanwhile, the prevalent fungus at the class level, Mortierellomycetes, was positively correlated with total phosphorus and available and total potassium, but negatively with alkaline-hydrolyzable nitrogen. Finally, the assignment of the functional guilds to the fungal operational taxonomic units (OTUs) revealed that the OTUs highly enriched in the downhill samples compared with the uphill samples, which were saprotrophs. Thus, this study is the first to report differences in the fungal community among the different soil/root samples and between *C. camphora* forests grown at different slope positions. We also identified the factors favoring the root-associated beneficial fungi in these forests, providing theoretical guidance for managing *C. camphora* forests.

## 1. Introduction

Plants are complex organisms associated with diverse microorganisms [1,2]. These microbes facilitate the mobilization and transportation of nutrients, prevent the occurrence of pathogens or pests, and reduce the effects of stress [3,4,5]. Plant-associated fungal communities profoundly impact plant health and productivity and ecosystem functions [6,7]. Therefore, a plant is not considered an independent entity but is a holobiont composed of the host and associated fungal populations; the holobiont forms the second genome of the host and is the evolutionary unit of selection [8,9,10]. A better understanding of the community assembly and the ecological interactions of plant-associated fungi will help identify strategies to improve agricultural productivity and enhance their practical application [11,12].

Soil is a complex dynamic environment with biological activities mainly controlled by microorganisms. Soil microorganisms boost plant nutrient availability by improving the bioavailability of nitrates, sulfates, phosphates, and other metals via nitrogen fixation and organic decomposition. In the soil, the role of fungi is exceptionally complex, and thus, they form the basis of soil ecosystems [13]. The most beneficial fungi play essential roles in soil nutrient cycling and plant development [14,15]. Although a few fungi are known to cause a range of plant diseases [16], many antagonize plant pathogens, decompose plant residues, provide nutrients, and stimulate plant growth. Specific fungi, such as the arbuscular mycorrhizal fungi, influence the composition of bacterial communities by directly altering the host physiology or indirectly altering the root exudation [17,18,19].

Root microorganisms that are closely related to plants are crucial among the various soil microorganisms. Certain root-associated fungi benefit the host by altering root development, increasing water and nutrient absorption, enhancing stress tolerance, and increasing plant biomass. At the same time, they obtain carbon and energy for survival from the plant root exudates and litter [20,21,22,23]. Studies have demonstrated that these rhizosphere microbes regulate plant morphology and physiology [24,25], promote plant growth and damage repair [26,27,28], increase biotic and abiotic stress tolerance [29,30], and improve plant ecological adaptability.

Various physical, chemical, and biological factors influence the colonization, abundance, and diversity of fungi in the rhizosphere and root endosphere. Soil texture and structure, organic matter content, macroaggregate stability, and other physical and chemical properties determine the habitable niche of fungi in the soil [31]. In addition, both biological and non-biological factors significantly affect the development and establishment of plant-microbial relationships, consequently influencing the net physiological effects on host plant growth. For example, environmental factors, such as topography, climate, vegetation, and human activities, significantly affect the spatial variability in soil properties and root-associated fungi communities in an ecosystem [32]. The slope, a major topographic factor, also affects microbial community structure and diversity by changing the microclimate, the physical and chemical properties of soil, and vegetation [33]. However, there is a lack of understanding of the correlation among slope, soil, and root-associated fungal communities.

The camphor tree (*Cinnamomum camphora*; Lauraceae) is an evergreen tree species distributed across the tropical and subtropical regions of Asia, especially China, Japan, Korea, and Vietnam [34]. It is used in wood processing and interior decoration and has high medicinal and economic value [35]. Studies on *C. camphora* mainly focused on essential oil composition, seedling technology, and secondary metabolites. A few researchers have analyzed *C. camphora*-associated microbial communities. For example, Kharwar et al. studied the endophytic fungal community in *C. camphora* and found that the mature tissues had more endophytic diversity than the young tissues [36]. Meanwhile, He et al. found that a few selected abundant endophytic fungi significantly influenced the decomposition process and the carbon and nutrient cycling in *C. camphora* forest [37]. However, the changes in the composition, diversity, and function of soil- and root-associated fungal communities of *C. camphora* under different slope positions and other site conditions remain unclear.

Moreover, the research on the analysis of fungal community structure in different niches of *C. camphora* forest by high-throughput sequencing technology has not been reported yet, and high-throughput sequencing based on ITS1 technology can reflect the colony structure characteristics of culturable and non-culturable fungi in samples to a greater extent and explore their diversity. Therefore, we used ITS1 high-throughput sequencing technology to analyze the fungal diversity and community composition in the bulk soil, rhizosphere, and root endosphere of *C. camphora* at different slope positions. We further assessed the effects of the physical and chemical properties of soil, soil enzyme activities, and microclimate on fungal communities and their functions. This study will deepen our understanding of the soil- and root-associated fungal communities of *C. camphora* and provide theoretical and practical guidance for sustainably managing *C. camphora* forest.

## 2. Materials and Methods

### 2.1. Experimental Procedure

The study was conducted in a five-year-old unharvested *C. camphora* forest in the Banlin State-owned Forest Farm at Anxi, Fujian Province. The study area was an artificial planted forest. Six test sample plots (24°56′39″–24°57′3″ N, 117°58′46″–118°0′20″ E; altitudes between 718 and 823 m) were selected, of which three were uphill, and three were downhill; a medium slope, a slope between 25–30 degrees, and the elevation difference between the uphill and the downhill sample plots was about 100 m. Each plot was 10 m × 10 m and contained 60 *C. camphora* plants. The region has a subtropical monsoon climate, with a hot and rainy climate in summer and a mild and humid climate in winter. The region had an average annual temperature of 18–19 °C and an average annual rainfall of 1600–1800 mm. The ground diameter, breast diameter, tree height, crown width, and total biomass of *C. camphora* plants in all plots were measured (Table S2).

The *Cinnamomum camphora* plants used in the study were in the summer shoot growth phase in June, and the sampling time was set as early June 2020. For each experimental plot, an agricultural environment monitoring instrument was used to measure the various forest microclimate factors, including air temperature (AT), water content (WC), soil temperature (ST), illumination (ILL), dew point temperature (Td), relative humidity (RH), altitude (ALT), and air pressure (ap). The soil from the 0–20 cm layer was collected using the five-point and quartering method, stored in a vacuum-sealed bag, and taken to the laboratory to determine the physical and chemical properties after air drying. The soil’s physical properties, including soil bulk density (SBD), mass water content (MWC), maximum field capacity (MFC), least field capacity (LFC), and total soil porosity (TSP), and the soil chemical properties, including pH, available (AK) and total potassium (TK), available (AP) and total phosphorus (TP), and alkaline-hydrolyzable nitrogen (AN), were determined in this study. The activity of four soil enzymes, including soil-urease (S-UE), soil-sucrase (S-SC), soil-catalase (S-CAT), and soil-acid phosphatase (S-ACP), were also measured. The bulk soil, rhizosphere, and root samples for the analysis were collected from the 0–20 cm soil layer. Three trees were selected from each sample plot (a total of 54 samples). Large pieces of soil attached to the roots were gently shaken off, and the roots were cut for immediate pretreatment. Finally, all the samples were transported to the laboratory at low temperature in a foam box filled with ice bags and stored in a −80 °C freezer for microbiota DNA extraction and high-throughput sequencing.

### 2.2. Sample Preparation and DNA Extraction

The rhizosphere soil attached to the soil was collected by eluting with phosphoric acid buffer solution [38] as follows: The roots were placed in 25 mL of phosphate buffer solution (6.33 g NaH_2_PO_4_·H_2_O, 16.5 g Na_2_HPO_4_·7H_2_O, 200 μL Silwet L-77 L^−1^; pH 7.0) in a 50 mL sterile centrifuge tube and vortexed for 15 s at the maximum speed on a vortex oscillator (Vortex-Geneie 2, Kezhida Information Technology Co., Ltd., Beijing, China). The suspension obtained by oscillation was filtered through a 100 μm sterilized nylon mesh filter into a new centrifuge tube and centrifuged at 3200× *g* for 15 min. The supernatant was removed, and the precipitate was collected as the rhizosphere soil and stored at −80 °C. The total DNA was extracted from the rhizosphere soil sample with the TGuide S96 Magnetic Soil DNA Kit (Tiangen Biotech Co., Ltd., Beijing, China). The DNA concentration was measured using the Qubit dsDNA HS Assay Kit on a Qubit 4.0 Fluorometer (Invitrogen, Thermo Fisher Scientific, Waltham, MA, USA), following the manufacturer’s instructions.

### 2.3. ITS1 Gene Amplification and Sequencing

The fungal ITS1 region was amplified from all DNA samples using the ITS1F (5′-CTTGGTCATTTAGAGGAAGTAA-3′) and ITS2 (5′-GCTGCGTTCTTCATCGATGC-3′) primers [39]. The PCR reaction mixture (10 μL) included 50 ng of genomic DNA, 0.3 μL of each primer, 5 μL of KOD FX Neo Buffer, 2 μL of dNTP (2 mM each), 0.2 μL of KOD FX Neo, and 10 μL of double-distilled water. The PCR conditions were as follows: an initial denaturation at 95 °C for 5 min, followed by 25 amplification cycles (at 95 °C for 30 s, 50 °C for 30 s, and 72 °C for 40 s), and a final extension at 72 °C for 7 min. The amplicon was purified using Agencourt AMPure XP Beads (Beckman Coulter, Indianapolis, IN, USA) and quantified using Qubit dsDNA HS Assay Kit on a Qubit 4.0 Fluorometer (Invitrogen, Thermo Fisher Scientific, Waltham, MA, USA). The purified amplicons were pooled in equal quantities and used to construct the libraries for sequencing on an Illumina Novaseq 6000 platform.

### 2.4. Sequence Analysis

The raw sequencing reads were spliced using the FLASH (v1.2.11) software [40] and filtered using the Trimmomatic (v0.33) software [41]. The chimeras were removed using the UCHIME v8.1 software [42] to obtain high-quality tag sequences. These sequences were then clustered using the USEARCH v10.0 software [43], with a similarity level of 97%; the filtering threshold of operational taxonomic units (OTU) was 0.005% [44]. Further, based on the Unite database (version 7.2, http://unite.ut.ee/index.php accessed on 10 July 2022), a representative sequence of OTUs was annotated with an RDP classifier (v2.2) [45,46] (80% confidence interval), and 1500 sequences were screened for subsequent analysis. The original sequence obtained by sequencing has been uploaded to the NCBI SRA database under the BioProject number PRJNA889716.

### 2.5. Statistical Analyses

All statistical analyses were performed using the R (v4.2.1) software. QIIME scripts were used to plot and calculate alpha diversity index including abundance-based coverage estimator (ACE), Shannon index, and Simpson index. Permutation multivariate analysis of variance (PERMANOVA) was performed using the vegan package to compare and determine the significant differences in beta diversity among the samples. The diversity in fungal community structure was studied using the Bray–Curtis differential method. The unconstrained principal coordinate analysis (PCoA) was used to visualize the fungal community structure. Meanwhile, the analysis of variance (ANOVA) method was used to assess the differences in the relative abundance of species among the different sample types (*p* < 0.05). Highly differentiated OTUs [47] were statistically analyzed using edgeR software. In addition, a redundancy analysis (RDA) was performed using the vegan package to analyze the relationship between *C. camphora* forest habitat and fungal community and was visualized using the ggplot2 software package.

Finally, the Fungi Functional Guide (FUNGuild) [48] was used to assign the functional guilds for the fungal community. According to nutritional status, fungi were classified into three major classes: pathotroph, symbiotroph, and saprotroph. These three main classes were divided into 12 subclasses, and a database guild was constructed to compare fungal functions.

## 3. Results

### 3.1. Distinct Fungal Communities in Root Endosphere and Rhizosphere

**Figure 1:**
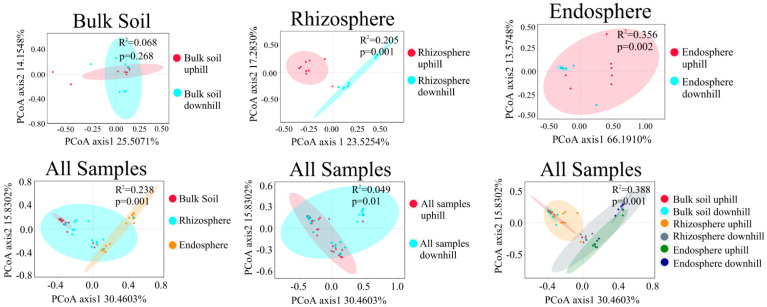
Unconstrained PCoA plot showing the variations in fungal communities based on the Bray–Curtis distance (PERMANOVA tested the significant differences). Ellipses indicate 95% confidence intervals for each sample type.

**Figure 2:**
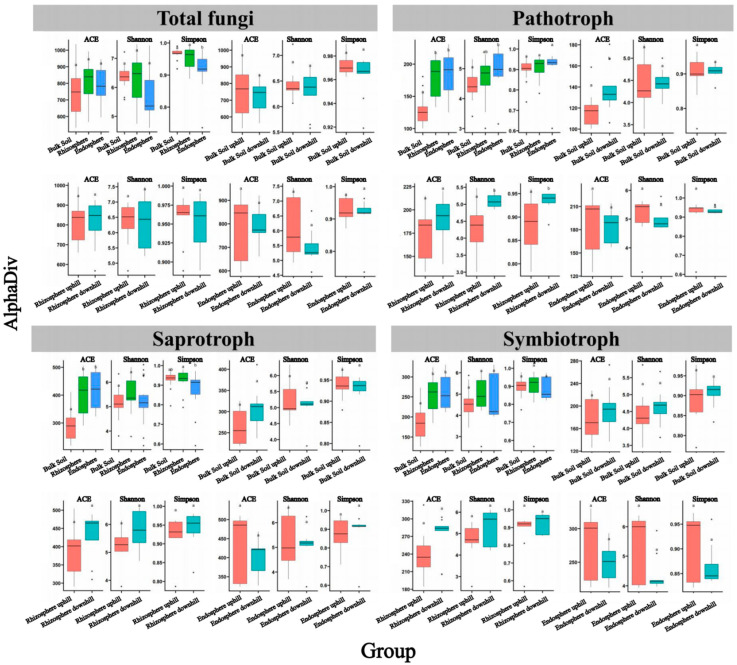
Alpha diversity indices of total fungi, pathotroph, saprotroph, and symbiotroph in the root endosphere, rhizosphere, and bulk soil samples. Abundance-based coverage estimator (ACE), Shannon index, and Simpson index are shown. Different lowercase letters indicate significant differences at *p* < 0.05.

Unconstrained PCoA showed significant differences in beta diversity among the fungal communities in the bulk soil, rhizosphere, and root endosphere but only minor differences between the uphill and downhill samples. Significant differences were detected in the rhizosphere and root endosphere fungal community composition between the uphill and downhill sites (*p* < 0.05); however, no significant difference was observed in the bulk soil samples (Figure 1). A further analysis revealed significant differences in the alpha diversity of fungal communities among the different sample types. The alpha diversity of pathotroph, saprotroph, and symbiotroph in the rhizosphere and root endosphere fungal communities was significantly higher than that in the bulk soil. The alpha diversity in the rhizosphere and bulk soil of the downslope sites was relatively higher than that of the upslope sites. In contrast, no significant difference was observed in the alpha diversity of the fungi in the root endosphere between the uphill and downhill samples (Figure 2).

### 3.2. OTUs in Root Endosphere and Rhizosphere

**Figure 3:**
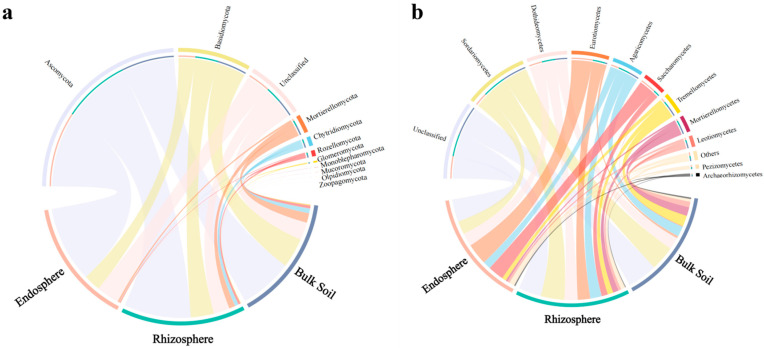
Relative abundance of fungi at the phylum (**a**) and class (**b**) levels in the various samples.

The relative abundances of fungi at different taxonomic levels were significantly different among the bulk soil, rhizosphere, and root endosphere of *C. camphora* forest. At the phylum level, the most abundant fungi were Ascomycota, followed by Basidiomycota and Mortierellomycota. Among them, Ascomycota was the predominant one in the root endosphere (63.91%). The proportions of Basidiomycota and Mortierellomycota in the bulk soil and rhizosphere were significantly higher than those in the root endosphere, which also had relatively low proportions of Chytridiomycota and Rozellomycota. Meanwhile, the proportion of unclassified fungi in the root endosphere was significantly higher (19.12%) than that in the bulk soil and rhizosphere (Figure 3a, Table S1). Taxonomic analysis at the class level indicated that the enrichment of the Ascomycota community in the rhizosphere and root endosphere was mainly influenced by a subset of classes, predominantly Eurotiomycetes and Saccharomycetes (Figure 3). ANOVA performed for the fungi at the phylum level in the bulk soil, rhizosphere, and root endosphere revealed a significantly higher relative abundance of Basidiomycota and Mortierellomycota in the bulk soil and rhizosphere than that in the root endosphere. Meanwhile, Ascomycota was the most abundant fungus in the root endosphere, consistent with the above results (Figure S1).

**Figure 4:**
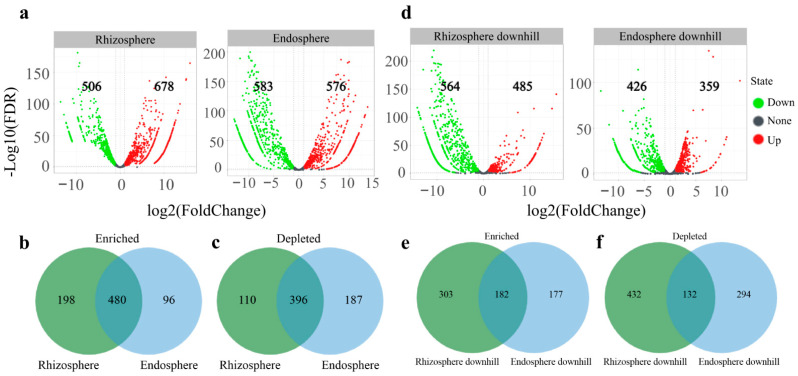
The enriched and depleted OTUs in the root endosphere, rhizosphere, downhill endosphere, and downhill rhizosphere. (**a**) Bulk soil was used as the control for differential abundance analysis. Enriched and depleted OTUs of the rhizosphere and endosphere compared with the bulk soil. (**b**) The number of differentially enriched OTUs between the root endosphere and rhizosphere. (**c**) The number of differentially depleted OTUs between the endosphere and rhizosphere. (**d**) Uphill rhizosphere and the root endosphere were used as controls for differential abundance analysis. Enriched and depleted OTUs of the rhizosphere downhill and endosphere downhill compared with the uphill rhizosphere and the root endosphere. (**e**) The number of differentially enriched OTUs between the downhill rhizosphere and endosphere. (**f**) The number of differentially depleted OTUs between the downhill rhizosphere and endosphere. Each point represents an individual OTU; the position along the X-axis represents the abundance fold change compared with bulk soil, and the Y-axis represents the −log 10 (FDR) obtained by correcting the *p*-value of the significant difference. The closer the point to the top of the graph, the more significant the difference is.

We further found that 678 and 576 OTUs were significantly enriched in the rhizosphere and root endosphere, respectively, compared with the OTU count of the bulk soil (Figure 4a). There were some overlaps in the differentially enriched and depleted OTUs between the root endosphere and rhizosphere. We classified these enriched OTUs into three subcommunities. The first subcommunity was classified as fully-enriched rhizosphere OTUs (198 OTUs) and was defined as fungi significantly enriched in the rhizosphere sample, distinguishing this sample type from the bulk soil. The second subcommunity was designated as fully-enriched root endosphere OTUs (96 OTUs) if fungi were significantly enriched in the root endosphere sample, distinguishing this sample type from the bulk soil. The third subcommunity designated OTUs coenriched in the root endosphere and rhizosphere (480 OTUs), as defined by OTUs enriched in root endosphere and rhizosphere samples, distinguishing these samples from the bulk soil (Figure 4b).

In addition, 506 OTUs were significantly depleted in the rhizosphere and 583 in the root endosphere compared with the bulk soil. Most of the OTUs depleted in the rhizosphere were also considerably depleted in the root endosphere (Figure 4c). Then, we analyzed the differences in the abundance of fungi in the rhizosphere and root endosphere samples from uphill and downhill. The analysis revealed that 485 OTUs were significantly enriched, and 564 were significantly depleted in the downhill rhizosphere compared with the uphill rhizosphere. Meanwhile, 359 OTUs were significantly enriched, and 426 were significantly depleted in the root endosphere of the downhill plots compared with the root endosphere of the uphill plots (Figure 4d). A total of 182 OTUs were enriched, and 132 were depleted in both the rhizosphere and the root endosphere of the downhill plots (Figure 4e,f).

### 3.3. Influence of Cinnamomum camphora Forest Habitat on Fungal Community

Furthermore, we measured the soil physical properties of *C. camphora* forest, including SBD, MWC, MFC, LFC, and TSP, and measured the pH, AN, TP, AP, TK, and AK content in the uphill and downhill plots. The AP, TP, AK, and TK content significantly differed between the upper and lower slopes, whereas pH and the measured soil physical properties did not (Table S3).

**Figure 5:**
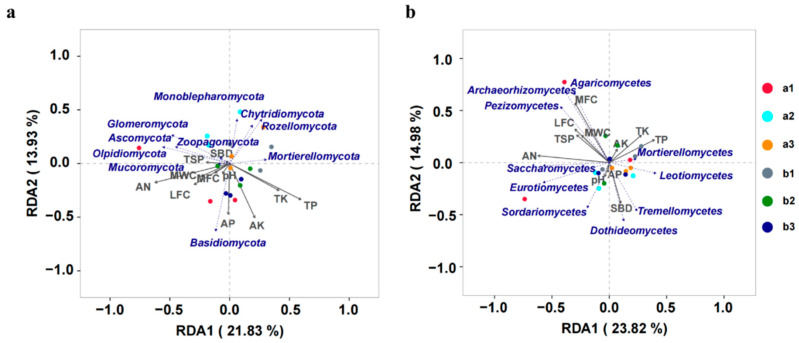
Redundancy analysis (RDA) ordination plots demonstrate the relationship between soil physical and chemical properties and fungal communities at the (**a**) phylum and (**b**) class levels. The arrows indicate the length and angle between the explanatory and response variables and reflect their correlations. Different samples are marked in different colors. a1, a2, and a3 represent the bulk soil samples of the uphill plots, and b1, b2, and b3 represent the bulk soil samples of the downhill plots.

Redundancy analysis (RDA) based on Bray–Curtis distance showed that soil physical and chemical properties, including pH, AN, AP, TP, AK, TK, SBD, MWC, MFC, LFC, and TSP, might have significant effects on the dominant fungal communities at the phylum level. The fungal species responded strongly to the soil’s physical and chemical properties. The dominant fungal phyla Mucoromycota positively correlated with SBD, TSP, MWC, AN, MFC, and LFC and were closely associated with each other. Basidiomycota positively correlated with pH, AP, and AK, whereas Mortierellomycota positively correlated with TP and TK (Figure 5a).

The dominant fungal communities at the class level varied significantly with changing physical and chemical soil properties. The dominant fungal class Mortierellomycetes was positively correlated with AK, TP, and TK but negatively with AN. Sordariomycetes had a significant positive correlation with pH and AP. Pezizomycetes, Archaeorhizomycetes and Agaricomycetes were positively correlated with TSP, LFC, MWC, and MFC but negatively with SBD (Figure 5b).

We measured the S-ACP, S-CAT, S-SC, and S-UE in the bulk soil and the microclimate factors of *C. camphora* forest in the uphill and downhill plots. The results showed that the S-ACP, S-CAT, and S-UE activities in the down-slope plots were significantly higher than those in the uphill plots, whereas no significant difference was observed in S-SC between the uphill and downhill plots (Table S4, Figure S2). The downhill plot had a lower altitude but higher dew point temperature and air pressure than the uphill plots, whereas no significant difference was observed in the other microclimate factors (Table S5, Figure S3).

Subsequent RDA showed that soil enzyme activity and microclimate factors significantly affected the fungal community. At the phylum level, the dominant fungi Ascomycota, Mucoromycota, Zoopagomycota, and Monoblepharomycota showed a significant positive correlation with S-SC, WC, ALT, and RH. Mortierellomycota was positively correlated with S-ACP, AT, AP, and ST, whereas Rozellomycota and Chytridiomycota were positively correlated with S-UE, S-CAT, and ILL (Figure S4a).

Soil enzyme activities and microclimatic factors also significantly affected fungal communities at the class level. The dominant fungus Mortierellomycetes was positively correlated with RH, ILL, AP, ST, S-UE, and S-CAT but negatively with WC and ALT. Meanwhile, Tremellomycetes, Dothideomycetes, and Leotiomycetes were positively correlated with AT and S-SC but negatively with S-ACP, Td, and RH (Figure S4b).

### 3.4. Changes in Predictive Functions of Fungal Communities

**Figure 6:**
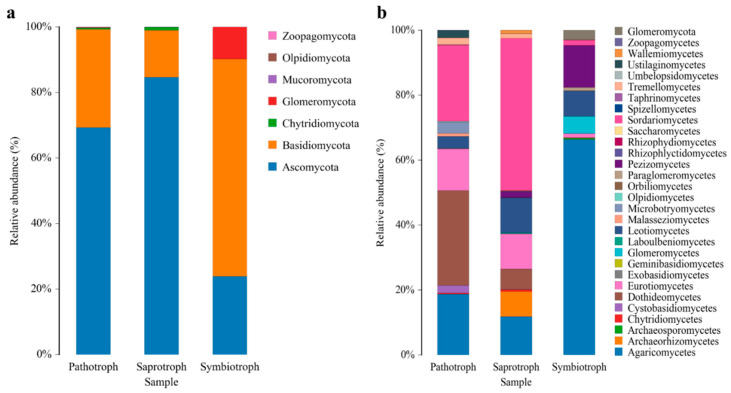
Relative abundance of the pathotroph, saprotroph, and symbiotroph functional fungi at the phylum (**a**) and class (**b**) levels in the samples.

Finally, the FUNGuild tool was used to analyze the functional guilds of the fungal communities. The fungi of the bulk soil, rhizosphere, and root endosphere samples were primarily classified into pathotroph, symbiotroph, and saprotroph ecological guilds according to nutritional patterns. At the phylum level, only seven functional fungi, Ascomycota, Basidiomycota, Chytridiomycota, Glomeromycota, Mucoromycota, Olpidiomycota, and Zoopagomycota, were identified in these three classes. Among them, Ascomycota and Basidiomycota showed significantly higher functional abundance in all three ecological guilds (pathotroph, symbiotroph, and saprotroph; Figure 6a). At the class level, 30 fungal species were identified in these classes. Among them, Dothideomycetes showed a relatively high abundance in pathotroph, whereas Sordariomycetes and Agaricomycetes showed the highest abundance in symbiotroph and saprotroph, respectively (Figure 6b).

**Figure 7:**
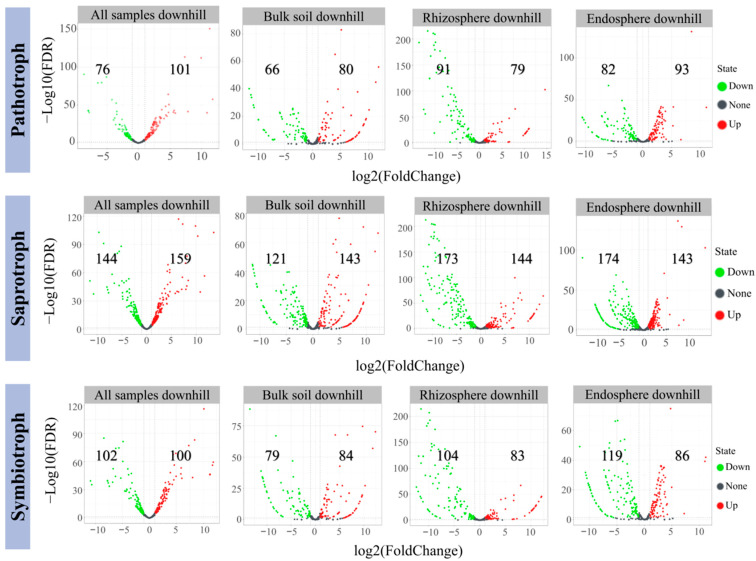
The enriched and depleted functional fungal OTUs in all downhill samples, downhill bulk soil, downhill rhizosphere, and downhill root endosphere. The enriched and depleted pathotroph, saprotroph, and symbiotroph are shown; all uphill samples, uphill bulk soil, uphill rhizosphere, and within uphill root endosphere were used as controls.

Furthermore, we found that the OTUs highly enriched in the downhill samples compared with the uphill samples were saprotrophs (159 OTUs), and the highly depleted OTUs were also saprotrophs (144 OTUs). Meanwhile, the number of saprotrophs significantly enriched and depleted in the downhill bulk soil, rhizosphere, and root endosphere compared with the fungal OTUs in the bulk soil, rhizosphere, and root endosphere of the uphill plots was significantly higher than that of the pathotroph and symbiotroph (Figure 7).

## 4. Discussion

### 4.1. Community Composition and Diversity of C. camphora Root-Associated Fungal Microbiome

The present study found that the alpha diversity of the pathotroph, saprotroph, and symbiotroph fungal communities was significantly higher in the rhizosphere and root endosphere than in bulk soil [49] (Figure 2). Plant roots secrete various low-molecular carbon sources; the fungal community consumes these components, creates favorable ecological niches, and promotes fungal diversity and richness [50]. In the absence of root plants, nutrient limitation and specific climatic conditions may hinder the growth of fungi and impede their hyphal proliferation [51]. Thus, only fungal species highly specialized for this niche can be found [52].

Our results highlight that soil- and root-associated microbiota interacts with the host plant in a complex manner depending on environmental conditions. Slope position is an important topographic factor affecting microbial distribution by controlling ecological factors’ spatial and temporal distribution and their combinations [53]. In this study, we found that among the pathotroph, saprotroph, and symbiotroph, the alpha diversity of fungi in the downhill rhizosphere was relatively higher than that in the uphill samples; however, no significant difference was observed in the alpha diversity of the root endosphere communities between the uphill and downhill samples. This observation is consistent with the results on the bacterial community diversity of *C. camphora* [49]. The alpha diversity of fungi probably decreases with less favorable chemical composition of uphill soils; however, root endosphere appears to be a more important filter of the microbiota so that the changing soil properties on the topographic gradient could not notably define taxonomic composition of endosphere.

The study further detected Ascomycota and Basidiomycota as the predominant fungal phyla in the *C. camphora* forest. The relative abundance of Ascomycota was the highest (63.91%) in the root endosphere, whereas that of Basidiomycota was the highest (24.97%) in the bulk soil (Figure 3a, Table S1). The results are similar to those of other plant fungal microbiome studies, such as the grapevine species [54], indicating that the dominant fungi are similar across species. In addition, the study found that the enrichment of Eurotiomycetes and Saccharomycetes at the class level drove species composition of Ascomycota in rhizosphere samples, consistent with their rapid growth characteristics. Like bacteria, these enriched fungi quickly adapt to and use the carbon source in the rhizosphere and occupy the niche [55]. 

A further analysis of the difference in OTU enrichment between the rhizosphere and root endosphere using bulk soil as the control showed that *C. camphora* forest supported considerably richer OTU communities in the rhizosphere than in the root endosphere (Figure 5). Meanwhile, there were fewer absent fungal OTUs in the rhizosphere than in the root endosphere. These observations are probably because the rhizosphere forms a highly active transition zone between the root surface and the soil through root secretion, mucus produced by the root cap, and release of root cells, and provides an appropriate niche for the growth, development, and reproduction of microbial communities [56].

### 4.2. Relationship between Fungal Community and C. camphora Forest Habitat

Slope position determines the spatial heterogeneity of soil physical and chemical properties by controlling the soil-forming processes [57]. Moreover, different slope positions differ in environmental conditions, such as light, heat, water, and air, and soil physical and chemical properties and soil enzyme activities. The present study found that the soil AN, TP, AP, TK, and AK were significantly higher in the downhill plots than in the uphill plots. The S-ACP, S-CAT, and S-UE activities were also considerably higher in the downhill plots than in the uphill plots. Typically, soils uphill are shallower and have little moisture retention capacity and deeper water tables. This may lead to higher evapotranspiration rates and higher drought and overheating risks [58,59,60,61]. In contrast, downhill locations are cool and have wet habitats with lower solar radiation and evapotranspiration rates [62,63,64]. Zhu et al. found that the downslope locations are hotspots receiving lateral surface and subsurface recharge, most likely due to the relatively lower elevation [65].

The structure of the microbial community in the soil could alter ecological cycling of nutrients [66]. The results of RDA analysis of soil physicochemical factors and fungal community showed that the dominant fungus at the phylum level, Mucoromycota, was positively correlated with the soil characteristics that favor plant growth. Similarly, Mortierellomycota probably increased the content of TP and TK in soil. Meanwhile, Mortierellomycetes, the dominant fungus at the class level, were associated with the increased levels of AK, TP, and TK content, but lower AN. In addition, Sordariomycetes may have promoted the increase in soil pH and AP content. Additionally, the dominant fungi Ascomycota and Mucoromycota at the phylum level were associated with the increased S-SC, WC, ALT, and RH. Similarly, the study found that Mortierellomycota correlated with the content of S-ACP, AT, AP, and ST, whereas Mortierellomycetes at the class level correlated with RH, ILL, AP, ST, S-UE, and S-CAT.

Ascomycota is an important driver of carbon and nitrogen cycling [67,68,69,70], the potential roles may include soil stability against erosion, seasonal plant biomass decomposition, and direct interaction with plants as endophytes or pathogens that induce selective decomposition of plant tissues. Similarly, Mucoromycota exists in a fungus–plant symbiosis and plays an essential role as an ecosystem degrader [71]. Mortierellomycota participates in mineralizing soil organic matter, breaking down crop residues in soil, and organic fertilizers into organic matter [72]. Therefore, the content of TP and TK may increase with the abundance of Mortierellomycota. In addition, Mortierellomycetes are potent producers of extracellular chitinases, which may control pathogens that infect roots, similar to bacteria [73]. Sordariomycetes have a worldwide distribution, are mainly adapted to terrestrial taxa [74,75], and have been isolated as endophytes from various plants [76,77]. 8 Most Sordariomycetes are carrion organisms involved in decomposition and nutrient cycling [78,79]. However, amplicon analysis does not provide any direct information about the natural history or life cycle of the detected fungal species. Therefore, further studies using other technologies, such as fluorescence in situ hybridization, confocal laser scanning microscopy, metagenome, or metatranscriptome approaches, will be necessary to gain insight into direct or potential plant interactions with soil- and root-associated fungal communities. In the future, high-throughput culture techniques will be needed to verify the ecological function of fungal communities.

### 4.3. Analysis of Functional Genes of the Fungal Community

The assignment of functional guilds to the fungal OTUs showed that the number of functional fungi at the phylum level was significantly less than that at the class level. In addition, the predicted functional guilds were the same across the sample types, and the predicted functional fungal groups were the same. Pathotroph, symbiotroph, and saprotroph were the three important functional classes of the *C. camphora* forests. Further differential abundance analysis showed that most functional fungi with differential expression in the downhill samples were saprotrophs, and relatively few were pathotroph, compared with the uphill samples (bulk soil, rhizosphere, and root endosphere samples). Fungal endophytes are affected by terrain factors and environmental changes [80], so in terms of functional composition of fungal community, pathotroph, symbiotroph, and saprotroph may also be affected by slope position to a certain extent. Among them, saprotrophs obtain nutrition by degrading dead host cells, pathotrophs by damaging host cells, and symbiotroph by exchanging resources with host cells.

## 5. Conclusions

This present study used the ITS1 high-throughput sequencing technology to systematically study the differences in the composition and diversity of *C. camphora*-associated fungal communities among root endosphere, rhizosphere, and bulk soil collected from two different slope positions. We found significant differences in beta diversity of the fungal communities of *C. camphora* forest among bulk soil, rhizosphere, and root endosphere. We identified Ascomycota and Basidiomycota as the dominant fungal phyla of the *C. camphora* forest. Ascomycota showed the highest abundance in the root endosphere, whereas Basidiomycota showed in the bulk soil. The alpha diversity of fungi in bulk soil and rhizosphere of *C. camphora* forest was relatively high in the downhill plots. Still, no significant difference was observed in alpha diversity in the root endosphere. Soil physical and chemical factors, soil enzyme activities, and microclimate factors substantially affected fungal communities in the *C. camphora* forest. The predicted fungal communities were similar in bulk soil, rhizosphere, and root endosphere.

Thus, this study is the first to report the differences in root endosphere, rhizosphere, and bulk soil fungal community structure, in addition to diversity and functional prediction of *C. camphora* forests at different slope positions, and also identified the factors favoring the root-associated beneficial fungi in these forests. Moreover, root endosphere appears to be a more important filter of the microbiota so that the changing soil properties on the topographic gradient could not notably define taxonomic composition of endosphere. This study provides a comprehensive understanding of soil- and root-associated fungal community diversity in different slope positions and a theoretical basis for rational utilization of these microbial resources in managing *C. camphora* forests. However, the dynamic changes in *C. camphora*’s microbial community during the growth phases need to be investigated.

## Data Availability

The original sequences obtained by sequencing have been uploaded to the NCBI SRA database; the BioProject number is PRJNA889716.

## Supplementary Materials

The following are available online at https://www.mdpi.com/article/10.3390/jof8111210/s1, Table S1: Mean relative abundances of top 10 differential phyla (%) in different sample types, Table S2: Growth index table of *C. camphora* in different plots, Table S3: Soil physical and chemical properties of different plots, Table S4: Soil enzyme activities of different plots, Table S5: The microclimate in the *C. camphora* forest of different plots. Figure S1: The X-axis represents species (the top 10 species with the lowest *p* values are shown); Y-axis represents the relative richness of species; columns with different colors represent samples; and * on the columns indicates that the difference is significant (*p* < 0.05). ** means the difference is very significant (*p* < 0.01), Figure S2: Soil enzyme activities, including soil acid phosphatase (S-ACP), soil catalase (S-CAT), soil sucrase (S-SC) and soil urease (S-UE), in the uphill and downhill sample plots of *C. camphora* were studied. Different letters indicated the significance at *p* < 0.05, Figure S3: The microclimate in the forest of *C. camphora* uphill and downhill sample plot, Figure S4: Redundancy analysis (RDA) ordination plots demonstrate the relationship between soil enzyme activities and forest microclimate and fungal communities at the (a) phylum and (b) class levels.
