# Supplementary figures and images for: Variation in Community Structure of the Root-Associated Fungi of Cinnamomum camphora Forest
Source: J Fungi (Basel). 2022 Nov 15;8(11):1210. doi: 10.3390/jof8111210 (PMC9699271; doi:10.3390/jof8111210)

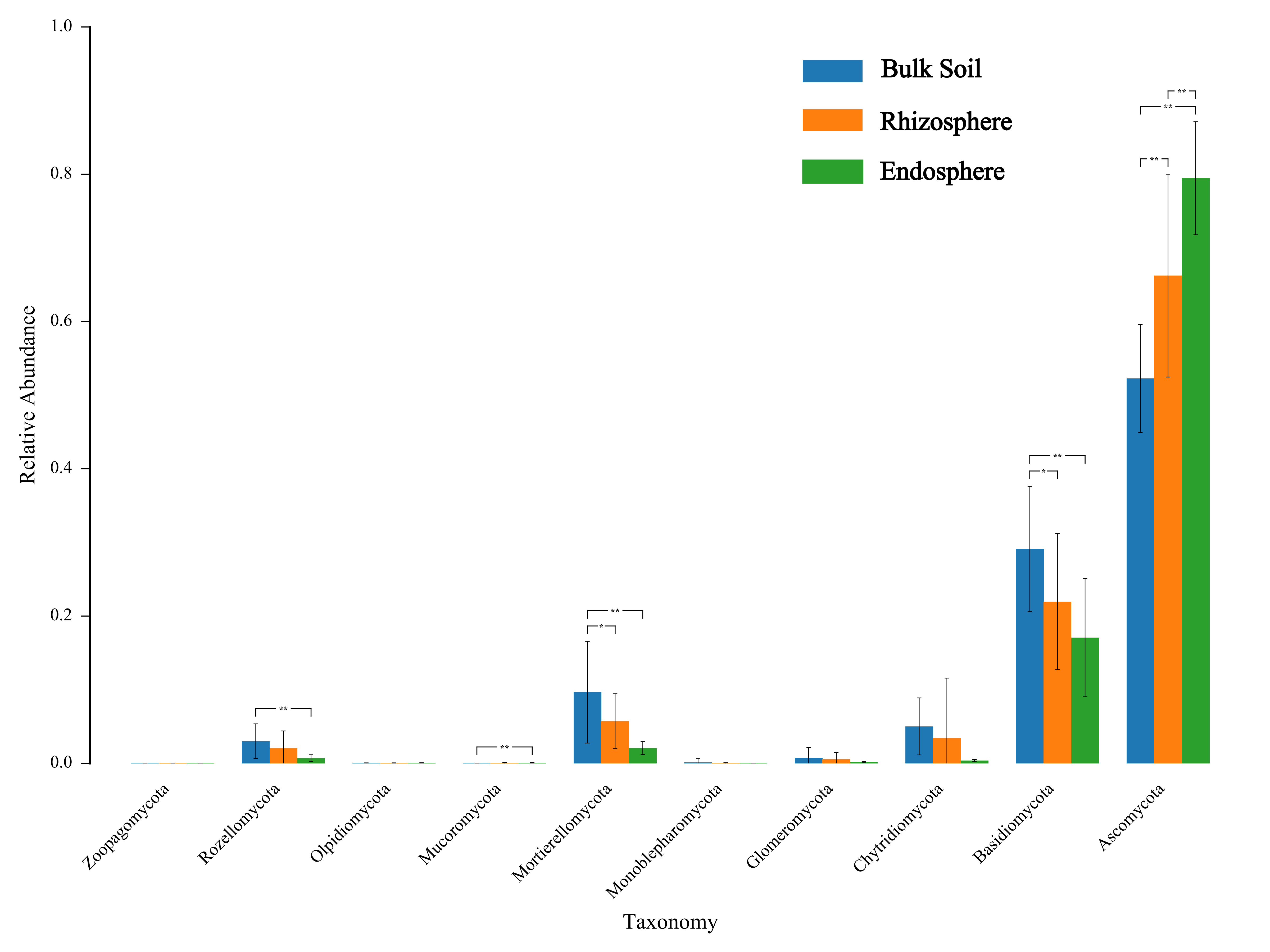

Supplement: Supplementary file 1 [file jof-08-01210-s001.zip › Supplementary Figure S1.png]

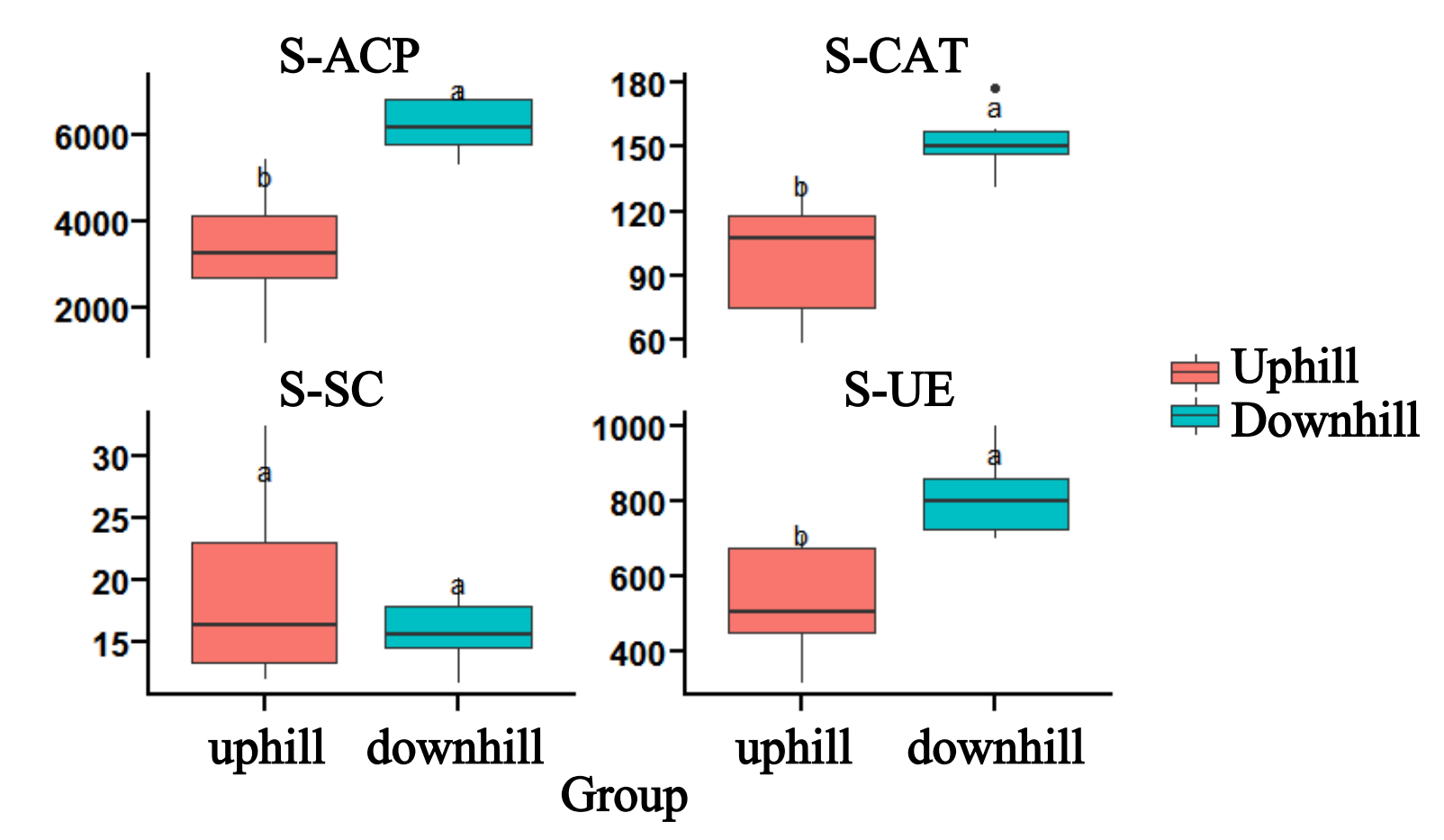

Supplement: Supplementary file 1 [file jof-08-01210-s001.zip › Supplementary Figure S2.png]

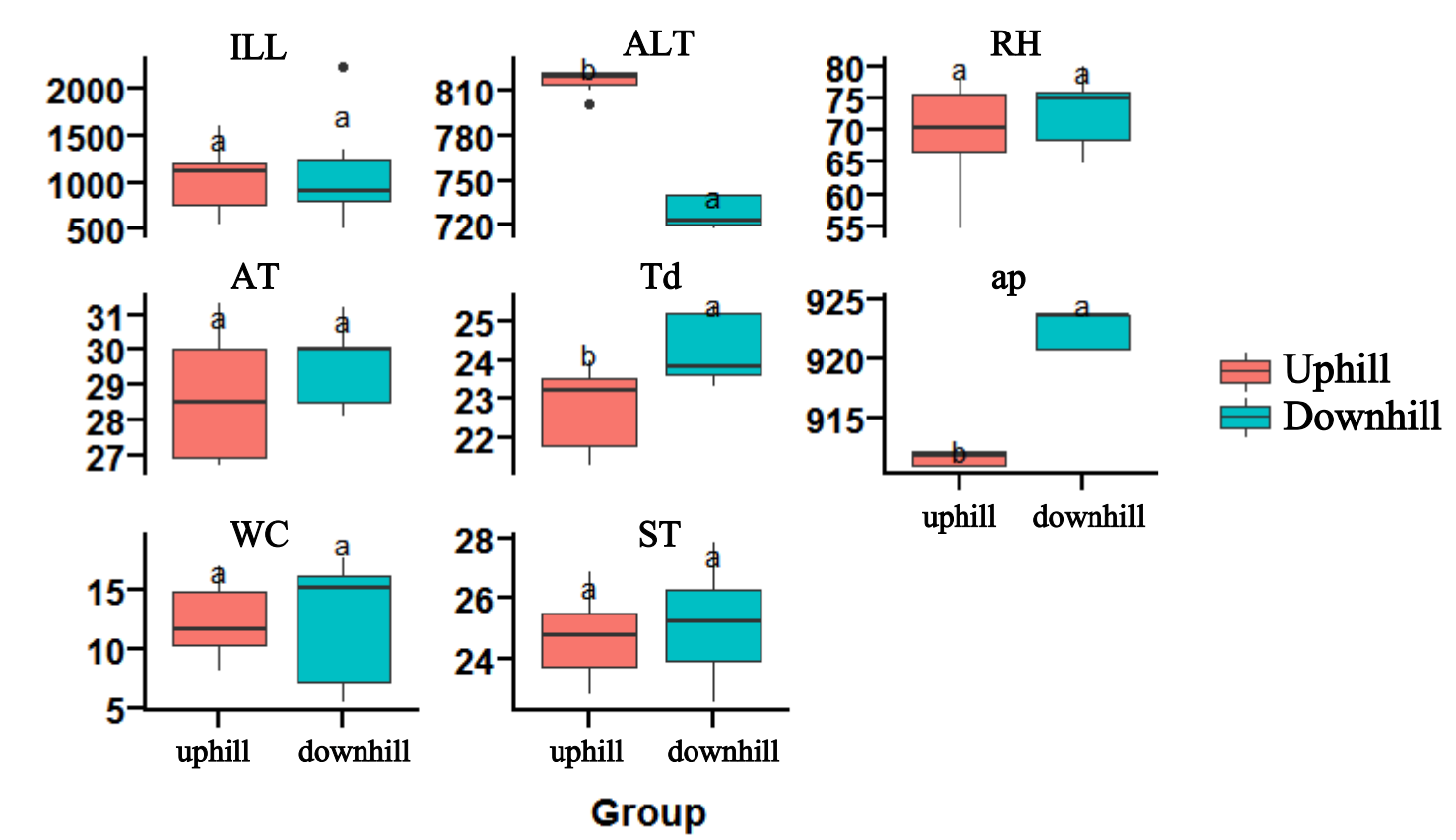

Supplement: Supplementary file 1 [file jof-08-01210-s001.zip › Supplementary Figure S3.png]

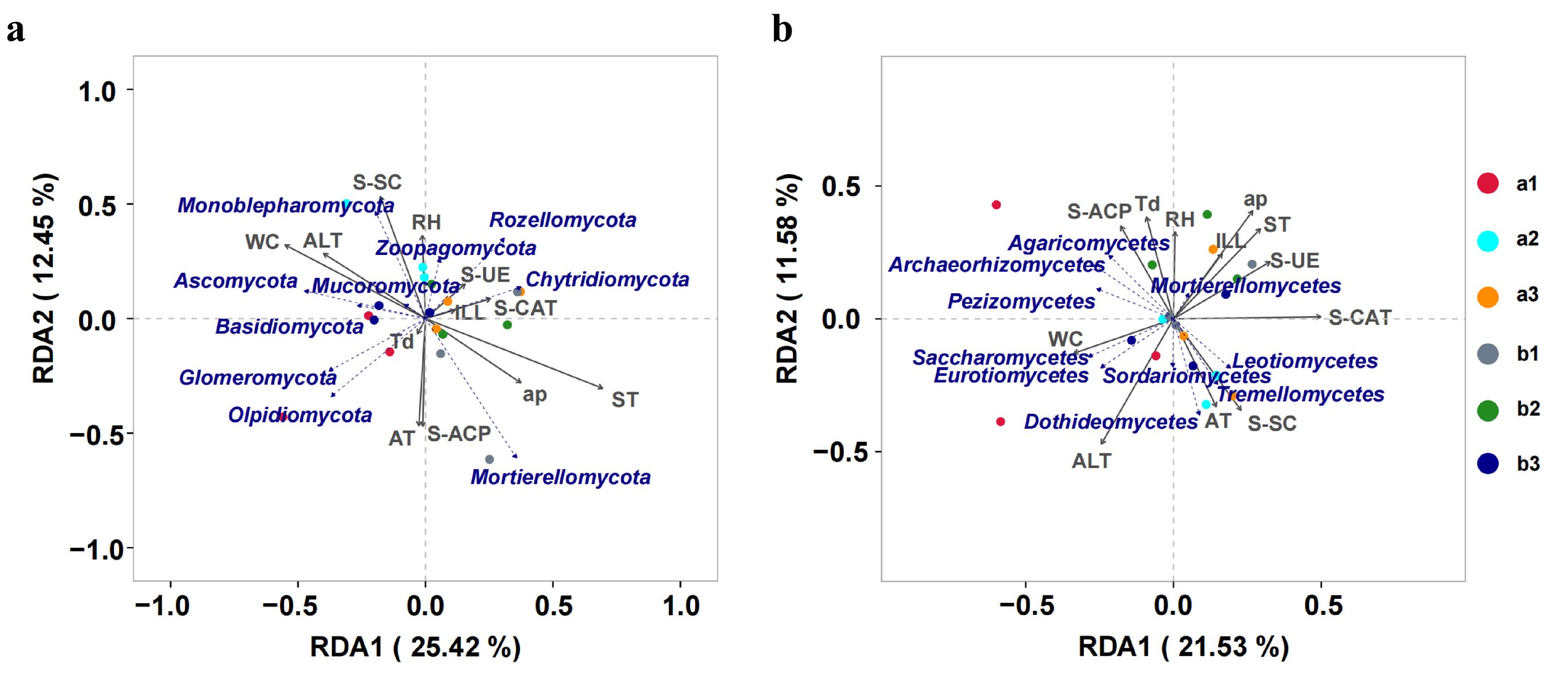

Supplement: Supplementary file 1 [file jof-08-01210-s001.zip › Supplementary Figure S4.png]
